# HIV outcomes within the context of orphans and vulnerable children programing: the 4Children project in South Sudan

**DOI:** 10.1186/s12879-022-07172-1

**Published:** 2022-02-24

**Authors:** Emily Coard, Daniel Oliver, Felix Monday

**Affiliations:** 1 Bloomberg School of Public Health, Johns Hopkins University, Baltimore, MD USA; 2 Catholic Relief Services, Baltimore, MD USA

**Keywords:** HIV/AIDS, Adherence, Viral load, South Sudan, Clinic records

## Abstract

**Background:**

Poor antiretroviral therapy (ART) adherence is a challenge to containing the spread of HIV. This is an especially difficult challenge in conflict and post-conflict settings. This study investigates the relationship between attendance in an Orphan and Vulnerable Children program in South Sudan and HIV-related outcomes, including clinic appointment attendance, frequency of viral load testing and viral load suppression rates.

**Methods:**

Patient records (n = 295) were selected from project-supported clinics in Juba, South Sudan, and analyzed to measure the association between enrollment status and select health outcomes. Data were collected at multiple time points between 2018 and 2019, to measure the strength of relationship between select treatment variables (e.g., viral load, retention in care, etc.). Given the structure of the data, non-parametric tests were applied to answer the research questions.

**Results:**

Analysis revealed three important trends: (1) enrollment in the 4Children project was associated with a statistically significant increase in the frequency of viral load testing; (2) there was an increase in median appointment attendance after program enrollment; and (3) there was improved management of viral load and CD4 count, albeit small, during the time period before and after enrollment.

**Conclusions:**

Data from South Sudan suggests that caregivers and children receiving project services saw improvement in treatment-related indicators. After enrolling in the project, overall amount of viral load testing increased from previous counts before enrollment. This suggests that after providing additional services with psychosocial and financial support to patients at the two hospitals in Juba, there was potential that similar interventions can support improved HIV outcomes.

## Background

After establishing independence in 2011, the Republic of South Sudan has experienced unstable healthcare and population displacement due to civil conflict [7, 8]. According to the CDC, the estimated HIV prevalence is 2.5% with 8000 AIDS deaths and 120,000 orphans due to AIDS in 2018 [16]. While prevalence is generally low, vulnerable populations, such as children, share a disproportionate part of the burden with mother-to-child transmission being one of the primary modes of infection [2, 8]. Additionally, the mobilization of military personnel, displacement of civilians, and high rates of movement from high-prevalence to low-prevalence areas combined with limited access to HIV care make the epidemic in South Sudan a growing concern [2].

To improve HIV health outcomes and prevent further transmission, regular access to anti-retroviral therapy (ART) and medical care is vital for adults and children with estimates of over 90% adherence needed for optimal viral load levels [7]. In a study based in Malawi by Kim et al. [11], risk factors including busy lifestyles, fear of stigma and poor emotional health impacted an individual’s ability to remain in care. These risk factors can become even more common in a country facing conflict and unstable infrastructure. There are support systems, however, that can help families maintain resilience during a variety of stressors [1]. HIV-related social support has been identified as one of the most common protective factors that facilitate treatment adherence [3]. Furthermore, financial security, in the form of stable employment and incomes have shown statistically significant relationships with treatment adherence [5, 15].

With a breadth of research to suggest support systems, such as psychosocial services from professionals and community lending programs, positively influence ART adherence, the Coordinating Comprehensive Care for Children (4Children) program designed HIV-treatment services incorporating these factors to deliver quality care to vulnerable children and caregivers. 4Children was a six year program, funded by the United States Agency for International Development, designed to improve the health and well-being of vulnerable children affected by HIV and AIDS and other adversities. In South Sudan, 4Children was implemented between 2015 and 2019. 4Children implemented a unique approach to health services for children and adolescents, offering individuals and families from high-density, low income neighborhoods in Juba, services that addressed challenges along the HIV treatment continuum. Services included: (1) group-based training and home visits to provide caregivers support, (2) group-based training, outreach-service delivery,^1^ and information campaigns to provide at-risk adolescents with life skills and health services, (3) Savings and Internal Lending Communities (SILC) groups to strengthen economic stability amongst families and individuals, and (4) training and other services such as community mapping,^2^ vulnerability assessments and activity monitoring^3^ targeted at building social welfare capacity for social workers in relevant communities.

This study examines HIV outcomes within the context of Orphans and Vulnerable Children (OVC) programming. Using project and clinic data, the study was designed to assess the potential for improved quality of care when enrolled in OVC programs. Studies such as these are uncommon in conflict and post-conflict settings. Data can be difficult to collect when issues of the safety and security of patients and clinic staff are at a premium [4]. The 4Children project was fortunate to have developed close relationships with each of the facilities associated with study, thus providing both the reassurance that safety came first, and that data, when collected, was done so accurately and with due protections in-place.

Three questions guided this study:

*Research question 1*: After enrollment in 4Children, did children and caregivers experience any differences in frequency of viral load testing compared to before enrollment?

Routine virologic monitoring is critical in HIV treatment and used as a benchmark by medical providers for treatment efficacy in adult and pediatric cases [9]. However, in low- and middle-income countries, viral load (VL) testing capabilities can be limited and CD4 count is often used instead [9]. In South Sudan, facilities have limited testing capabilities. Both CD4 and viral load testing are used as the standard of care. Testing occurs during follow-up visits, every 6 months for high VL patients, and 12 months for low VL patients. As 4Children sought to optimize outcomes through improved ability to utilize long-term healthcare, the program analyzed data on the number of times each patient had their VL and/or CD4 measured before the program and how many times each patient had each measured after the program began. This allowed project staff to determine if routine monitoring changed.

*Research question 2*: After enrollment, is there a difference in child and caregiver retention in care compared to before the program?

Appointment data was collected to understand if additional support such as training and financial assistance could improve retention in care. The program recorded the number of appointments scheduled and the number of appointments attended and calculated attendance rates from the results.

*Research question 3*: What differences, if any, did participants have as it concerns HIV outcomes before enrollment as compared to after enrollment?

In order to understand health outcomes, data on CD4 counts and VL were classified into categorical variables to assess participants before and after enrollment in the program. CD4 counts of 500 and above are considered to be in the normal range while viral load copies/ml 1000 and less are considered virally suppressed. Participants with CD4 counts less than 500 or viral load copies/ml greater than 1000 were classified as at-risk. While there are differing opinions as to the utility of these values, particularly as it concerns the use of CD4 counts, there remains utility within resource limited settings [6]. Further, South Sudan uses a CD4 < 500 as a threshold level [14, 17].

## Methods

### Data collection

Using a data capture tool, 4Children staff collected data from child and adult clinic records. Records were included if the patient was enrolled in the 4Children program, had voluntarily disclosed their HIV-positive status and had accessed care at either Juba Teaching Hospital or Juba Military Hospital. Program staff compiled a list of all enrolled participants who met these criteria (387 adult and 84 child participants). Adult and child participants were not necessarily related. Adult participants were randomly selected from this list to provide the necessary size to conduct reliable statistical tests, based on a standard sample size calculation. This resulted in the collection of 211 adult records. Given the smaller size, data from all 84 child records were collected.

Upon identifying the list of participants to be included in the analysis, data on HIV testing, appointments, viral load, and CD4 count in addition to demographics variables were collected. While CD4 percentages were considered for younger participants, these data were not relevant to this study population as all children under age 5 received viral load tests. Data were collected at three timepoints: pre-intervention, 6 months after enrollment, and 12 months after enrollment. The “Pre-intervention” time period varied, depending on patient appointment attendance, ranging from 12 months to several years prior to enrollment. Unique identifiers were assigned to de-identify all participants. Final data collection and reports did not contain names or other personally identifiable information.

### Statistical analysis

All data were entered into and analyzed using Stata 16.0. Symmetry tests were used to understand the differences in distribution before and after. As the data were non-parametric and non-symmetric, the sign test was chosen to compare the continuous variables of HIV appointment attendance rate and frequency of viral load and CD4 count testing before and after the 4Children program. Assumptions of equal variances were not met and thus the Wilcoxon signed rank test was not performed in favor of a sign test. Categorical baseline and follow-up risk measurements were compared using the McNemar test to understand any difference in dichotomous risk levels for participants. The McNemar test was run comparing the most recent test result in the program with baseline measurements. All tests were additionally used to analyze differences between age categories of under 18 and 18 years old and over.

## Results

### Study population

Participant ages ranged from 1 to 61 years (mean = 29.97, SD = 14.23) with 28.5% under 18 years, 70.5% 18 years and older, and 1% who did not identify their age. 71.2% identified as female, 27.5% identified as male, and 1.3% did not have their sex recorded. 57% of those in the program received treatment and services at Juba Military Hospital while 43% received treatment and services at Juba Teaching Hospital. Additionally, 95% of participants in the 4Children program had already begun treatment for HIV before enrollment and 5% started treatment after enrollment.

Before enrollment, 59.1% of participants had received testing for viral load or CD4 count. Out of participants who received testing, 43.1% tested for CD4, 16.6% tested for viral load. More than 40% received no test. After enrolling in the 4Children program, 81.7% of participants were tested and had a median viral load of 403 (IQR = 24,557)) at their first viral load test after enrollment. In the time before enrollment, a mean of 8.76 (SD = 7.43) appointments were scheduled for participants and a mean of 8.40 (SD = 7.51) appointments were attended. From the date of 4Children enrollment to the present, a mean of 12.99 (SD = 11.19) appointments were scheduled and a mean of 13.34 (SD = 11.54) appointments were attended.

### Frequency of testing

The one-sided test indicated no significance for the hypothesis that median testing frequency was greater before program enrollment (p-value = 1.00), however, median testing frequency appeared to be significantly higher after the participants were enrolled (p-value = 0.000). With a one-sided sign test yielding a p-value of 0.00, the null hypothesis that there was no difference between testing frequency medians was rejected. With more negative signs (156 observed signs) than positive signs (83 observed signs), the results suggest a difference in frequency of VL and CD4 count testing after participants enrolled in the program. As a result, these data suggest a positive, statistically significant relationship between enrollment in the 4Children project and an increase in the frequency of testing. Finally, sign tests were run on age categories under and over 18 years. The two-sided test for 18 years and over yielded a p-value of 0.0049, indicating there was a difference after 4Children enrollment for this age group. Additionally, the two-sided test for under 18 years indicated a difference between before and after (p-value = 0.00) (see Table 1).

**Table 1:** Summary statistics—frequency of testing

	Observations	Mean	Standard deviation	Median	IQR
Before	295	1.03	1.19	1.00	2.00
≤ 18	84.00	1.17	1.32	1.00	2.00
> 18	211.00	0.97	1.13	1.00	1.00
After	295.00	1.45	0.95	2.00	1.00
≤ 18	84.00	1.58	1.01	2.00	1.00
> 18	211.00	1.39	0.92	1.00	1.00

### Retention in care

One-sided tests show that the appointment attendance after enrollment appears to be greater than the attendance rate before. A one-sided Sign Test for appointment attendance in all age groups resulted in a p-value of 0.00. Consequently, the test indicates an increase in median attendance after program enrollment and a possible improvement for participants in regard to their ability to attend appointments related to managing their HIV care (see Table 2). Further two-sided sign tests for patients under 18 years retained statistical significance with a p-value of 0.00 indicating that missed appointments decreased for this age group after enrollment. Additionally, for patients 18 years and older, a significant value was retained for improved appointment attendance (p-value = 0.00) (see Tables 3 and 4).

**Table 2:** Sign test—testing frequency

Sign	All ages			≥ 18			< 18		
	Obs	Expected	*P*	Obs	Expected	*P*	Obs	Expected	*P*
Positive	83	119.5		68	87		14	32	
Negative	156	119.5		106	87		50	32	
Zero	56	56		34	34		20	20	
Total	295	295	0.00	208	208	0.0049	84	84	0.00

**Table 3:** Summary statistics—retention in care

	Observations	Mean	Standard deviation	Median	IQR
*Before*					
Scheduled	295	8.76	7.43	6.00	7.00
≤ 18	84	8.96	8.93	6.00	5.00
> 18	211	8.68	6.77	7.00	7.00
Attended	295	8.40	7.51	6.00	7.00
< 18	84.00	8.57	8.92	6.00	5.50
> 18	211.00	8.33	6.89	6.00	7.00
*After*					
Scheduled	295.00	12.99	11.19	9.00	12.00
≤ 18	84.00	12.37	12.10	9.00	10.00
> 18	211.00	13.24	10.82	10.00	13.00
Attended	295.00	13.34	11.54	10.00	12.00
≤ 18	84.00	13.46	13.09	9.00	11.00
> 18	211.00	14.24	18.02	10.00	13.00

**Table 4:** Sign test—attendance rate

	All ages			≥ 18			< 18		
Sign	Obs	Expected	*P*	Obs	Expected	*P*	Obs	Expected	*P*
Positive	9	37.5		4	21		5	16.5	
Negative	66	37.5		38	21		28	16.5	
Zero	220	220		166	166		51	51	
Total	295	295	0.00	208	208	0.00	84	84	0.00

### Risk categories

Using a McNemar test (see Tables 5 and 6), 29 participants were classified as at risk before the program started but transitioned to not at risk after enrollment. Additionally, 38 participants remained not at risk throughout the entire time, while 44 were classified as at risk before and after enrollment. Only 12 participants started out as not at risk before the program and progressed to not at risk after enrolling. The proportion of those at risk before and after the program were then examined by testing the null hypothesis:

$${\text{Ho: p}}_{{{\text{before}}}} = {\text{p}}_{{{\text{after}}}}$$

**Table 5:** Summary statistics—CD4 and VL counts

		Median	IQR	Min	Max
*Before enrollment*					
All ages	CD4	412	322	35	42,263
	VL	552	2990	0	205,132
≥ 18	CD4	379	241	35	851
	VL	344	3435	0	205,132
< 18	CD4	448	371	46	2703
	VL	1173	2492	0	49,642
*After enrollment*					
All ages	CD4	N/A	N/A	N/A	N/A
	VL	403	24,557	0	1,492,014
≥ 18	CD4	N/A	N/A	N/A	N/A
	VL	0	17,997	0	192,773
< 18	CD4	N/A	N/A	N/A	N/A
	VL	2370	30,395	0	1,492,014

**Table 6:** Classification table for McNemar analysis

	After enrollment	Before enrollment		Total	*P*
		At risk	Not at risk		
All ages	At risk	44	12	56	
	Not at risk	29	38	67	
	Total	73	50	123	0.0079

	After enrollment	Before enrollment		Total	*P*
		At risk	Not at risk		
≥ 18	At risk	29	6	35	
	Not at risk	22	28	50	
	Total	51	34	85	0.0025

	After enrollment	Before enrollment		Total	*P*
		At risk	Not at risk		
< 18	At risk	15	6	21	
	Not at risk	7	10	17	
	Total	22	16	38	0.7815

The test resulted in a chi statistic of 7.05 and was statistically significant with a p-value of 0.0079, indicating increased management of VL and CD4 count due to a lowered proportion of participants in the at risk category. However, it is important to note the relative difference was − 0.34 with a confidence interval of − 0.63 and − 0.05, which indicates that there may have only been a small change between the proportion categorized at risk before and after program enrollment. Finally, the McNemar test for patients under 18 found no significant difference in risk category before and after enrollment (p-value = 0.7815). However, the McNemar test run on patients 18 years old and older produced statistically significant results for risk category differences (p-value = 0.0025). It is also important to note that the under 18 years old sample size was smaller than the ages 18 and over sample and this may have affected the results of the test once divided by age category.

## Discussion

The data analyzed as part of this study suggest that OVC programs can have important roles to play for caregivers and children affected by HIV/AIDS, particularly within post-conflict settings. Consistent VL testing for HIV patients has been identified as a key tool for medical providers to track and manage a patient’s progression in status and virologic control [9, 12]. After enrolling in the 4Children program, the amount of VL testing increased from counts before enrollment. This suggests that after providing services, including psychosocial and financial support, participants exhibited improved outcomes. While the precise mechanisms driving this change are beyond the scope of this paper, these findings add to the growing body of evidence suggesting that OVC programs play an important role in epidemic control.

Reducing missed appointments has previously been studied as a means to improve healthcare outcomes and individual-level disparities in HIV [10, 13, 19]. After enrolling in the program, evidence suggest that the ability to attend appointments improved for some participants while it remained the same for another substantial portion of this population. It should be noted that that the standard of care in South Sudan dictates an average of 12 appointments per year (minimum 6, maximum 18). Various appointment frequency depends on the patient’s viral load measures. As multi-month dispensation of ARVs becomes more common, it can be expected that these appointments would reduce. However, at present, these data suggest that participants attended more appointments after enrolling in the intervention.

Participation in 4Children also appears to be associated with modest improvements in viral load suppression. While the change observed before and after program enrollment was small and attribution to the intervention was not measured, these findings point to the need for further, rigorous investigations. As viral load testing becomes increasingly accessible in Sub-Saharan Africa [18], there is greater ability to examine how different interventions can help children and their caregivers reach improved outcomes.

Ongoing studies examining OVC and HIV treatment programs will continue to explore the link between a variety of support services targeting children and caregivers to better understand circumstances for optimal HIV management. While the results of this analysis are not generalizable nor meant to establish causality, these findings offer preliminary evidence of promising interventions for low-resource and high-instability settings such as South Sudan.

### Limitations

The study faced four primary limitations. First, as enrollment in the OVC program did not use random assignment, the results from the study are not generalizable to a wider population. Enrollment in 4Children was voluntary and everyone who participated received the intervention. Second, the sample used for this study is not representative of the wider population. Participants were enrolled into the intervention based on an assessment of their vulnerability but also their location within select geographic areas. The sample size for patients under 18 was also small, which may have resulted in unreliable findings when tests were run on the two different age groups (under and over 18 years old). Third, health facilities only began collecting VL data in April 2017. As such, the study team was unable to do comparisons of viral load for all study participants, as some were enrolled before April 2017 and baseline measurements were collected in CD4 counts. Consequently, data from participants were combined into risk categories rather than using the values of the test results. Fourth, potential limitations to the data could also include the difference between scheduled appointments and attended appointments. Many participants differed in counts between scheduled and attended. This might be due to follow-up appointments generated by the initial scheduled appointments or to participants requesting more appointments. While these are plausible explanations, this could not be confirmed for every participant.

## Conclusions

The purpose of this study was to explore the relationship between OVC programming and HIV outcomes, all within the context of post-conflict South Sudan. As discussed, important outcomes were achieved during the implementation of this project, including: (1) increased frequency of viral load testing; (2) increased appointment attendance; and (3) modest improvements in viral load suppression. While this study was not designed to measure the impact of the intervention, it helps lay the foundation for future research concerning the direct effect of OVC programming on health outcomes, particularly those occurring within post-conflict settings.

## Data Availability

The dataset generated and analyzed for this study are available at 10.5061/dryad.qjq2bvqgj.

## Abbreviations

AIDS: Acquired immunodeficiency syndrome
ART: Antiretroviral therapy
CDC: Centers for Disease Control and Prevention
HIV: Human immunodeficiency virus
OVC: Orphans and vulnerable children
SILC: Savings and Internal Lending Communities
VL: Viral load

## Footnotes

1 Outreach-service delivery refers to services that are provided through field agents at the participants home or community.

2 Community mapping is used to document the assets available within a community (e.g., health facilities, social services, etc.).

3 Activity monitoring is a results-based management activity whereby project staff assess activity delivery and its quality.
